# Silica Nanoparticles as the Adjuvant for the Immunisation of Mice Using Hepatitis B Core Virus-Like Particles

**DOI:** 10.1371/journal.pone.0114006

**Published:** 2014-12-01

**Authors:** Dace Skrastina, Ivars Petrovskis, Ilva Lieknina, Janis Bogans, Regina Renhofa, Velta Ose, Andris Dishlers, Yuri Dekhtyar, Paul Pumpens

**Affiliations:** 1 Latvian Biomedical Research and Study Centre, Riga, Latvia; 2 Institute of Biomedical Engineering and Nanotechnologies, Riga Technical University, Riga, Latvia; Academia Sinica, Taiwan

## Abstract

Advances in nanotechnology and nanomaterials have facilitated the development of silicon dioxide, or Silica, particles as a promising immunological adjuvant for the generation of novel prophylactic and therapeutic vaccines. In the present study, we have compared the adjuvanting potential of commercially available Silica nanoparticles (initial particles size of 10–20 nm) with that of aluminium hydroxide, or Alum, as well as that of complete and incomplete Freund's adjuvants for the immunisation of BALB/c mice with virus-like particles (VLPs) formed by recombinant full-length Hepatitis B virus core (HBc) protein. The induction of B-cell and T-cell responses was studied after immunisation. Silica nanoparticles were able to adsorb maximally 40% of the added HBc, whereas the adsorption capacity of Alum exceeded 90% at the same VLPs/adjuvant ratio. Both Silica and Alum formed large complexes with HBc VLPs that sedimented rapidly after formulation, as detected by dynamic light scattering, spectrophotometry, and electron microscopy. Both Silica and Alum augmented the humoral response against HBc VLPs to the high anti-HBc level in the case of intraperitoneal immunisation, whereas in subcutaneous immunisation, the Silica-adjuvanted anti-HBc level even exceeded the level adjuvanted by Alum. The adjuvanting of HBc VLPs by Silica resulted in the same typical IgG2a/IgG1 ratios as in the case of the adjuvanting by Alum. The combination of Silica with monophosphoryl lipid A (MPL) led to the same enhancement of the HBc-specific T-cell induction as in the case of the Alum and MPL combination. These findings demonstrate that Silica is not a weaker putative adjuvant than Alum for induction of B-cell and T-cell responses against recombinant HBc VLPs. This finding may have an essential impact on the development of the set of Silica-adjuvanted vaccines based on a long list of HBc-derived virus-like particles as the biological component.

## Introduction

Although vaccines offer the most cost-effective solution to prevent infectious and possibly non-infectious diseases, the rate of success in the vaccine development is currently declining [1]. The reviving of vaccinology depends on the well-balanced improvement of two basic components of vaccines: the biological component and the adjuvant. As the adjuvant is critical for the augmentation of the proper immunogenicity of the biological component, which obviously is not able to induce strong humoral and cellular immune responses, the biological component therefore requires the assistance of an adjuvant [2]. While the current progress in the elaboration of numerous biological vaccine components is obvious [2], [3], the scope of safe and efficient adjuvants for human use is rather limited and needs an urgent expansion [2].

The existing vaccine adjuvants are comprised of different classes of substances including mineral salts, emulsions, and microorganisms-derived molecules [4]. Only two of them: a group of aluminium derivatives including aluminium phosphate, aluminium hydroxyphosphate, and aluminium hydroxide (Alum) and monophosphoryl lipid A (MPL) are licensed by the FDA for use in human vaccines today [5]. Alum was described by Alexander Glenny and co-authors more than 90 years ago [6], when they found that a suspension of the Alum-precipitated diphtheria toxoid had much higher immunogenicity than the fluid toxoid (for a review see [7]). Presently, Alum is the component of many popular vaccines against diphtheria, tetanus, pertussis, poliomyelitis, hepatitis A and B, rabies, anthrax, and others [8]. MPL, a TLR4 agonist, was licensed for human use in combination with Alum in a human papilloma virus vaccine [9].

However, application of Alum as the adjuvant is under controversial debate. Local reactions, swelling, indurations, cutaneous nodules and allergic reactions are found in the sites of Alum injections [10]–[12]. Moreover, adsorption of the biological component of vaccines to Alum may destabilise proteins [13]. By mechanism of action, Alum is able to augment the humoral immune response only; it is inefficient in the raising of cell-mediated immunity [7]. This disadvantage of Alum is surmounted now by its combination with monophosphoryl lipid A (MPL), which guarantees efficient induction of the cell-mediated immunity [14].

The rapid development of nanotechnology and nanomaterials raises new promises in the generation of novel adjuvant systems. Silicon dioxide (Silica) nanoparticles are one of the most favourable adjuvant candidates. The particles are solid with a diameter less than 100 nm. Adjuvanting capacities of different structures of Silica such as amorphous Silica [15], mesoporous Silica [16], and Silica nanorattles [17] have been investigated. Incorporation of recombinant interferon β from *E.coli* into mesoporous Silica particles induced antibody responses, which could be compared with the Alum- and incomplete Freund's adjuvant (IFA)-adjuvanted responses [16]. Mesoporous Silica demonstrated its adjuvanting capability by both intramuscular and oral routes of immunisation of mice with bovine serum albumin [18]. No changes in organ tissues were found by histopathological studies in mice, which had been immunised with ovalbumin and mesoporous Silica nanoparticles as an adjuvant [19]. Mesoporous Silica particles demonstrated a tuning effect on the development of the effector T cells by induction of cell-mediated anti-tumour immunity [20], [21]. Very recently, the adjuvanting effect of the Silica nanoparticles of 50 nm in diameter was demonstrated for the immunisation of mice with capsomeres of the recombinant virus-like particles (VLPs), namely, murine polyomavirus particles [22].

Here, we evaluated the Silica nanoparticles as the possible adjuvants for the immunisation of mice with one of the most studied VLP models, recombinant hepatitis B virus (HBV) core (HBc) particles, which possess high proper immunogenicity on B-cell, T-cell, and cytotoxic T lymphocyte (CTL) level (for review see [23]). Today, the recombinant HBc particles from *E.coli* are available at high levels of synthesis and quality standards [24] and may be regarded as promising scaffolds for a large set of prophylactic and therapeutic vaccines [3], [25], [26]. After formulation with HBc VLPs, Silica nanoparticles with initial 10–20 nm diameters in solid state appeared as large complexes, which rapidly precipitated from the solution. Although Silica demonstrated lower efficiency of the HBc adsorption than Alum, both adjuvants provided similar levels of humoral anti-HBc response, characteristic IgG2a/IgG1 ratios of anti-HBc antibodies, and the ability to improve the specific T-cell response in combination with MPL.

## Materials and Methods

### Production, purification, and characterisation of Hepatitis B core virus-like particles

HBc VLPs (HBV320 genome, genotype D, subtype *ayw2* [27]) were produced and purified as previously described [24]. Briefly, transformed *E.coli* K802 cells producing HBc VLPs (wet weight 8 g) were resuspended in 4 volumes of the lysis buffer (50 mM Tris-HCl, pH 8.0, 5 mM EDTA, 150 mM NaCl, 50 µM PMSF, 0.1% Triton X100) and ultrasonicated at 22 kHz 8 x for 10 s. After clarification of lysate at 10,000×g for 30 min, soluble proteins were precipitated with ammonium sulphate at 10% saturation, at 4°C for 1 h, followed by centrifugation at 10,000×g for 30 min. VLPs in the supernatant were precipitated with ammonium sulphate at 35% saturation, at 4°C overnight, followed by centrifugation at 10,000×g for 30 min. The sediment was dissolved in 20 mL of PBS buffer with 0.5 M urea and 50 µM PMSF, and subjected to anion exchange chromatography on a Sepharose Q High Performance (GE Healthcare, Sweden) XK26/20 column using AKTA Avant chromatography system (GE Healthcare, Sweden). Elution was performed with buffers A (20 mM NaH_2_PO_4_/Na_2_HPO_4_, pH 7.3, 0.3 M NaCl) and B (20 mM NaH_2_PO_4_/Na_2_HPO_4_ pH 7.3, 1 M NaCl) until 100% of B in 300 mL at flow rate of 5 mL/min. Peak fractions were pooled and concentrated with 500 kDa MidGee Hollow Fiber Cartridge. Concentrated VLPs were subjected to size exclusion chromatography on a Sepharose 4 Fast Flow (GE Healthcare, Sweden) 120 mL column (16×600 mm) at a flow rate of 0.5 mL/min. The peak fractions were analysed using SDS-PAGE, native agarose gel electrophoresis, dynamic light scattering (DLS), and electron microscopy (EM). DLS analysis was performed on a Zetasizer Nano ZS instrument (Malvern Instruments Ltd, UK), in line with previously described VLP measurements [28]. The results were analysed by DTS software (Malvern, version 6.32). For the EM analysis, materials were adsorbed on Formvar carbon-coated copper grids, materials containing HBc VLPs were stained with 1% aqueous uranyl acetate solution for 2 min. The grids were examined with a JEM-1230 electron microscope (JEOL Ltd., Japan) at an accelerating voltage of 100 kV.

### Adjuvants

The Silica nanopowder, with the particle size of 10–20 nm, surface area (BET, Brunauer, Emmett and Teller theory) 140–180 m^2^/g, 99.5% purity, were purchased from Sigma-Aldrich (USA). Distilled water was used to prepare stock solutions of 20.0 mg/mL before immunisation. The stock solution was sterilised with UV irradiation for 2 h. An Alum adjuvant Alhydrogel was purchased from Brenntag Biosector (Denmark). Complete Freund's adjuvant (CFA) and Incomplete Freund's Adjuvant (IFA) were supplied by Statens Seruminstitut (Denmark).

For adsorption experiments, 0.125 mg of the HBc VLPs in PBS were taken and combined with 0.5, 1.25, 2.5 or 5.0 mg of Silica or Alhydrogel in an Eppendorf tube and normalised with PBS to the final volume of 1.0 mL. The suspensions were gently mixed by overhead rotation at room temperature for 2, 24 or 48 h. For analysis of non-adsorbed HBc VLPs, the samples were centrifuged at 3,000×g, at room temperature for 20 min and protein concentration in the supernatants was measured by a NanoDrop analyser (Thermo scientific, ND-1000, USA) at 230 and 260 nm. The HBc VLPs concentration was calculated by a formula: C (µg/mL)  = 183×OD_230nm_–75.8×OD_260nm_ The percentage of the adsorbed VLPs was calculated against a control sample of HBc VLPs in PBS, without the added adjuvant.

For DLS and EM analysis, 0.125 mg of the HBc VLPs and 1.25 mg of the appropriate adjuvant separately or in combination were used and normalised with PBS to the final volume of 1.0 mL and incubated at room temperature for 2 h. For DLS, the supernatants were analysed after centrifugation at 3,000×g, at room temperature for 20 min; for EM, samples were used without centrifugation.

### Mice and immunisation

Pathogen-free, female BALB/c mice between 6-8 weeks of age were obtained from the Latvian Experimental Animal Laboratory of the Riga Stradins University and maintained under pathogen-free conditions in accordance with the principles and guidelines of the Latvian and European Community Laws. Five animals were used in each experimental group. The experimental protocol was approved by the local Animal Protection Ethical Committee of the Latvian Food and Veterinary Service (permission no. 55/15.03.2013).

Mice were injected intraperitoneally (i.p.) or subcutaneously (s.c.) with 25 µg of HBc VLPs in PBS formulated with following adjuvants in a total volume of 0.2 mL (for normalisation using PBS): (a) 250 µg of Alhydrogel, (b) 250 µg of Silica, (c) with Complete Freund's adjuvant for the first injection followed by two injections with Incomplete Freund's Adjuvant (CFA/IFA) mixed with protein solution in 1∶1 ratio by volume, (d) with IFA for all three injections mixed with protein solution in 1∶1 ratio by volume and (e) with VLPs diluted in PBS. Animals were immunised on days 0, 14, 28 and bled on day 42. Mice were then sacrificed by cervical dislocation.

For the T-cell proliferation assay and cytokine test, groups of BALB/c mice with five animals in each group were immunised s.c. with 25 µg HBc VLPs formulated as before (a) with 250 µg of Alhydrogel, (b) with 250 µg of Silica, or (c) with 250 µg of Alhydrogel supplemented with 10 µg of MPL (InvivoGen, USA), (d) with 250 µg of Silica supplemented with 10 µg of MPL, and (e) with 10 µg of MPL only. On day 10, mice were bled for an ELISA test; they were then sacrificed, and lymph node cells were obtained.

### ELISA assays

For detection of anti-HBc antibodies, ninety-six-well plates (Nunc, USA) were coated with HBc VLPs (10 µg/mL in 50 mM sodium carbonate buffer, pH 9.6, 100 µL per well) at 4°C overnight. After blocking with 1% BSA in PBS at 37°C for 1 h, serial dilutions of mouse sera were added to the plates and incubated at 37°C for 1 h. After washing 3 times with PBS containing 0.05% Tween-20, 100 µL horseradish peroxidase conjugated anti-mouse antibody (Sigma, USA) at a 1∶10,000 dilution was added per well. Following incubation at 37°C for 1 h, plates were washed and substrate OPD (Sigma, USA) was added for colour development. Optical absorbance measurements were performed with Multiscan (Sweden) at 492 nm. The end-point titers were calculated as the highest serum dilution that resulted in an absorbance value exceeding three times that of the negative control (serum obtained from non-immunised mice).

An isotype-specific ELISA was performed for the detection of IgG1 and IgG2a subclasses of anti-HBc antibodies, using mouse monoclonal antibody isotyping reagent ISO2 (Sigma, USA), as secondary antibodies using peroxidase conjugate of monoclonal anti-goat/sheep IgG antibodies (Sigma, USA). The end-point titers were calculated as stated above.

### T-cell proliferation assay and cytokine test

Murine splenocytes were harvested using Red blood cell lysis buffer (Sigma, USA), lymphocyte suspensions were prepared (5×10^6^ cells/mL) and co-cultured with HBc VLPs (10 µg/mL) in RPMI 1640 medium (Gibco, Germany) at 37°C for 96 h in a humidified 5% CO_2_ atmosphere. 1 µCi of [^3^H]-thymidine (TdR, Amersham, USA) was added to each well for a final 18 h period of incubation, and [^3^H]-thymidine incorporation was measured by Liquid scintillation β-counter (Beckman, USA). The proliferative response was defined as a stimulation index (SI) calculated as the mean *cpm* corrected for background [^3^H]-thymidine incorporation in the absence of the HBc VLPs.

Cytokine levels in the medium of cell cultures from the wells were measured 48 h after the T-cell proliferation test was initiated using the BD OptEIA ELISA sets for Mouse IFN-γ and the Mouse IL-2 (BD Biosciences, USA), according to the manufacturer's instructions.

## Results

### Efficiency of Silica and Alhydrogel in HBc VLPs adsorption

**Figure 1:**
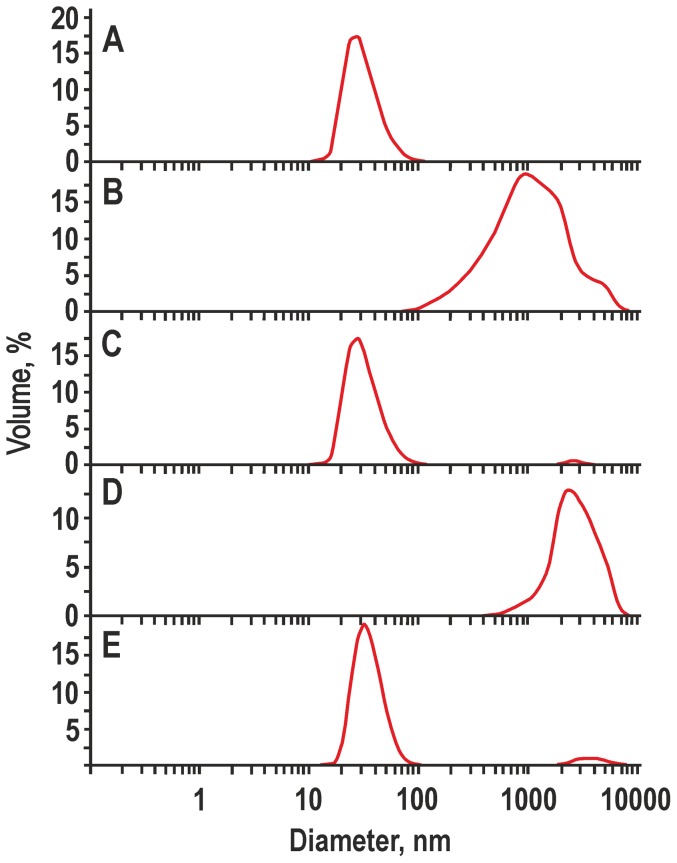
DLS analysis of the particle size distribution in the supernatants of adjuvanted HBc VLPs. **A** – HBc VLPs, **B** – Silica in PBS, **C** – Silica-adjuvanted HBc VLPs, **D** – Alhydrogel in PBS, **E** – Alhydrogel-adjuvanted HBc VLPs. The results of the DLS size distribution are shown with the particle diameter in nm on the x-axis and the number of particles in % on the y-axis.

The DLS analysis was performed to characterise the supernatants after low-speed centrifugation of suspensions of HBc VLPs before and after formulation with Silica and Alhydrogel (Fig. 1). Analysis of free, non-adjuvanted HBc particles revealed a peak at the 34 nm size (Fig. 1A), which is close to the expected 35 and 32 nm diameters of the T = 4 and T = 3 forms of HBc VLPs, respectively [29]–[31]. DLS of the free Silica and Alhydrogel, without added HBc VLPs, demonstrated the presence of a heterogeneous material dispersed between the interval of 100 nm and 10 µm, with a maximum at 1 µm, in the case of Silica (Fig. 1B) and between the interval of 1 µm and 10 µm, with a maximum at 2 µm, for Alhydrogel (Fig. 1D). After formulation of HBc particles with Silica (Fig. 1C) and Alhydrogel (Fig. 1E), the heterogeneous adjuvant-characteristic profile diminished to the utmost minimum with very small peaks at 2 µm and 3 µm of size for Silica and Alhydrogel, respectively. It was therefore assumed that addition of HBc particles to both Silica and Alhydrogel stimulated rapid sedimentation of the formulated material, which is in accordance with our previous observations [32].

**Figure 2:**
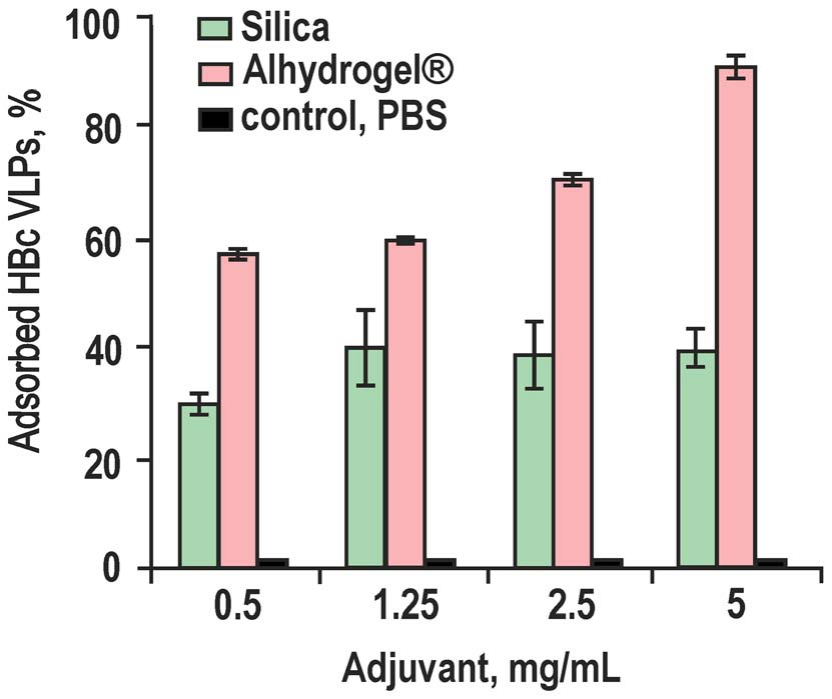
The adsorption efficiency of HBc VLPs at different concentrations of Silica and Alhydrogel. A portion of the 0.125 mg of HBc VLPs was formulated in a total volume of 1.0 mL with the appropriate concentration of adjuvant suspensions or with PBS as a control for 2 h at room temperature. The results are presented as the means ± standard deviation (SD) from three experiments.

To compare the adsorption capacity of Silica and Alhydrogel for HBc VLPs, the level of non-adsorbed HBc VLPs was measured spectrophotometrically in the supernatants of appropriate HBc/adjuvant-formulated probes for several doses of adjuvants (Fig. 2). Thus, for Silica, after 2 h of incubation, the adsorption of HBc VLPs, slightly depending on the dose, reached a plateau with 30–40% of the added HBc being adsorbed; extension of the incubation time to 24 and 48 h allowed to adsorb 60% of added HBc for the entire range of used Silica concentrations and further prolongation of the incubation time did not improve the adsorption level. However, for HBc VLPs adjuvanted by Alhydrogel the amount of the adsorbed HBc VLPs correlated directly with the dose of adjuvant and reached 92% at the highest dose.

**Figure 3:**
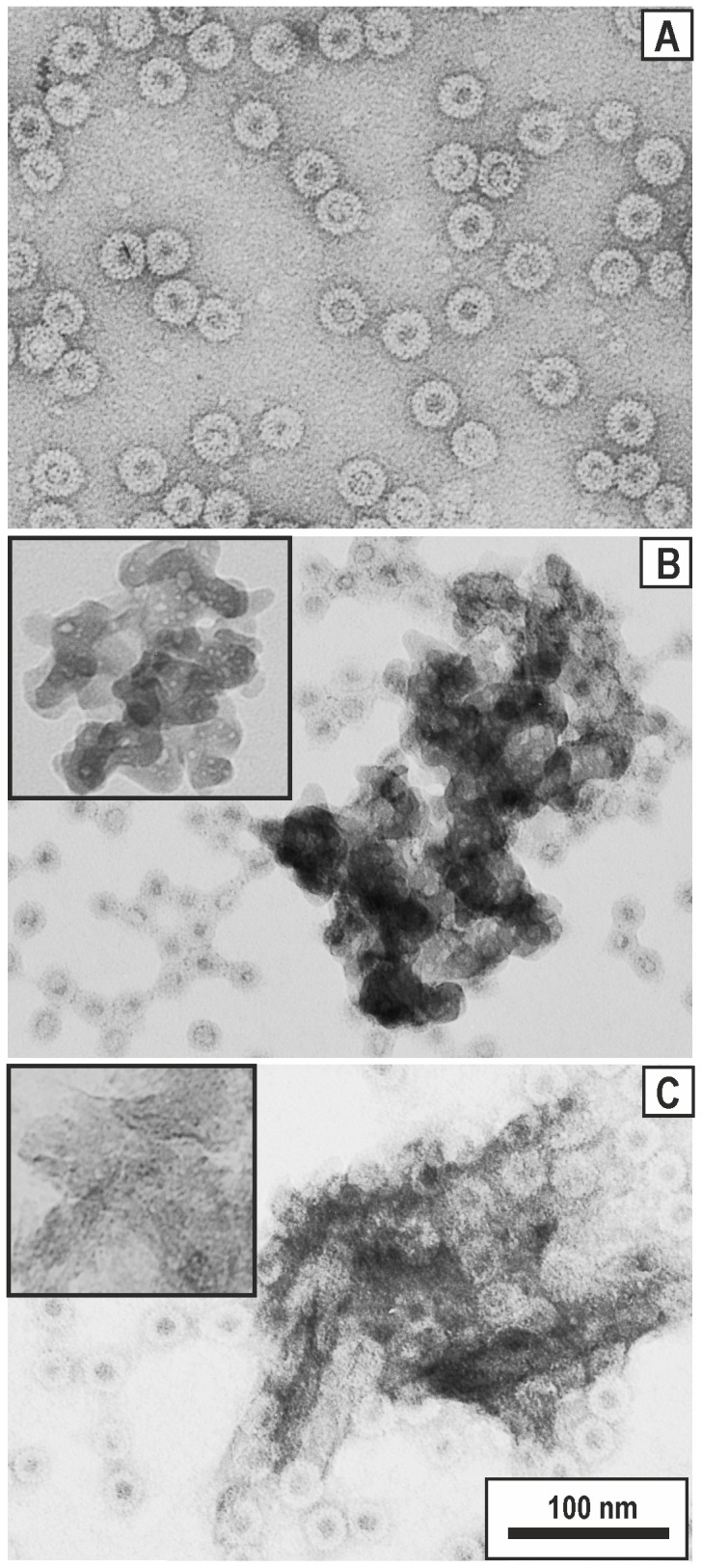
Electron microscopy of the HBc VLPs before and after formulation with adjuvants. **A** – HBc VLPs without adjuvant, **B** – Silica-adjuvanted HBc VLPs**, C** – Alhydrogel-adjuvanted HBc VLPs. EM pictures of the initial non-loaded Silica and Alhydrogel are shown on the appropriate insets. Scale bar, 100 nm.

To characterise components before and after the formulation, the EM analysis was performed (Fig. 3). The EM of the initial HBc VLPs (Fig. 3A) demonstrated separated spherically shaped particles with an expected 4∶1 ratio of 35 and 32 nm sized particles, respectively [33], [34]. Silica nanopowder suspension in PBS appeared as large aggregates (Fig. 3B, inset) similar in size to the aggregates formed by Alhydrogel (Fig. 3C, inset). After the formulation of HBc VLPs with Silica (Fig. 3B) or Alhydrogel (Fig. 3C), free VLPs along with large aggregates were observed in both cases, supporting the previous conclusion that only part of HBc VLPs is adsorbed by Silica and Alhydrogel.

### Adjuvanting effect of Silica on the HBc-induced humoral response in mice in comparison to Alum, CFA and IFA

**Figure 4:**
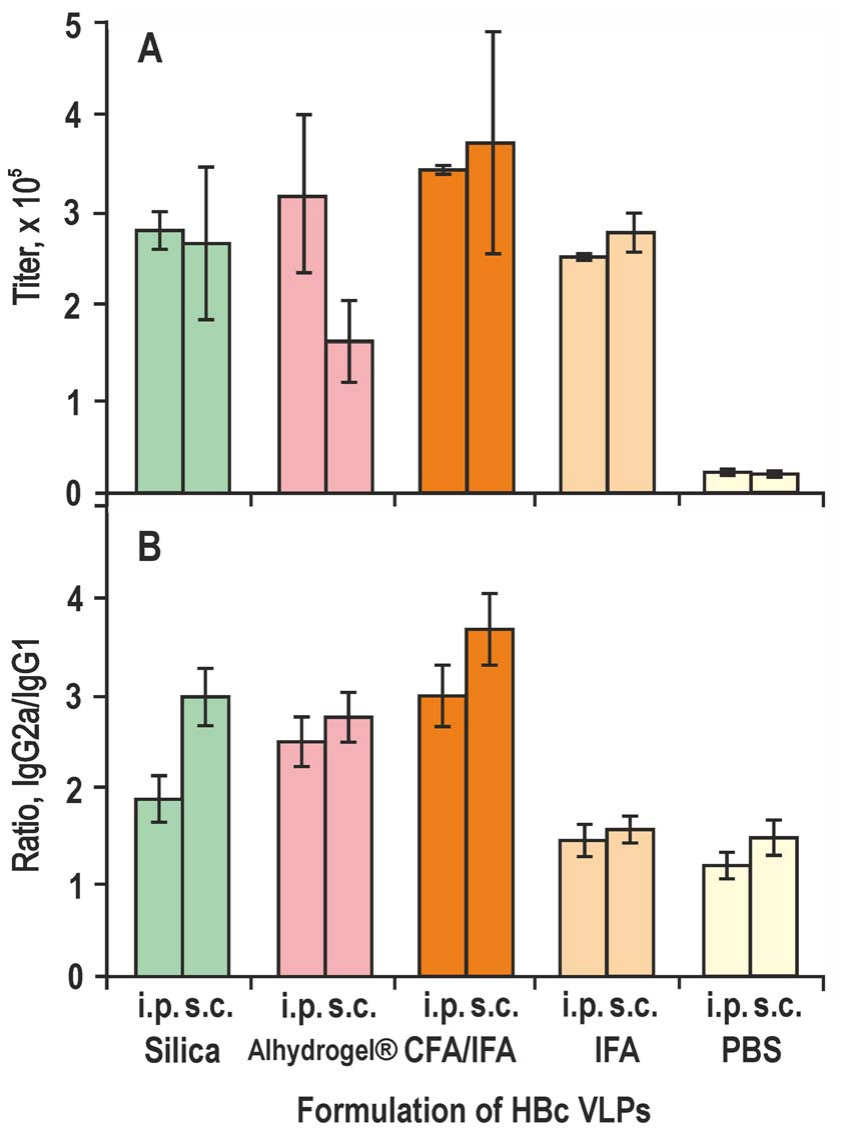
Induction of humoral anti-HBc response in BALB/c mice by HBc VLPs formulated with different adjuvants. **A** – total anti-HBc titers, **B** – ratio of anti-HBc IgG2a/IgG1 isotype titers. The HBc VLPs were adjuvanted with Silica, Alhydrogel, CFA/IFA, IFA or diluted in PBS. Mice were bled after intraperitoneal (i.p.) and subcutaneous (s.c.) immunisation at day 42 after the first injection. The results represent anti-HBc titers or ratios of the IgG2a/IgG1 titers as the means from five mice ± standard deviation (SD).

Intraperitoneal and subcutaneous immunisations of BALB/c mice were performed with HBc VLPs adjuvanted in parallel by Silica and traditional adjuvants Alhydrogel, CFA and IFA alone (Fig. 4). Immune responses were documented on day 42 after the first immunisation as shown in Fig. 4A. The adjuvanting effect of Silica was not found to differ markedly from that of the Alhydrogel when mice were injected by the intraperitoneal route with anti-HBc titers of 1∶279,300 and 1∶312,000 for Silica and Alhydrogel, respectively. By subcutaneous injection, the anti-HBc response induced by Silica-adjuvanted HBc was the same as in the case of intraperitoneal administration, exceeding 1.6 times of that induced by Alhydrogel-adjuvanted HBc with corresponding titers of 1∶265,500 and 1∶160,400, respectively. However, the highest anti-HBc titers were obtained for HBc adjuvanted by CFA/IFA with corresponding titers of 1∶340,000 and 1∶367,000 for intraperitoneal and subcutaneous injections, respectively. Use of IFA as the only adjuvant in all three injections elicited anti-HBc titers close to those obtained in Silica-adjuvanted immunisation with anti-HBc titers of 1∶250,000 and 1∶273,400 for intraperitoneal and subcutaneous injections, respectively. Anti-HBc titers for HBc diluted in PBS, without any adjuvant, was approximately 10 times lower after both intraperitoneal and subcutaneous injections.

Isotyping of the induced anti-HBc antibodies revealed a regular excess of IgG2a over IgG1 in all cases (Fig. 4B), which have been found to be an intrinsic immunological feature of the full-length HBc formed VLPs [24], [35], [36]; however, the level of the IgG2a/IgG1 ratio was clearly dependent on both the particular adjuvant and the administration route used for immunisation. Generally, subcutaneous administration induced a higher IgG2a/IgG1 ratio than the intraperitoneal administration. Thus, in the case of Silica-adjuvanted HBc, the IgG2a/IgG1ratio reached 2.0 and 3.0 for intraperitoneal and subcutaneous injections, respectively. Similarly, formulation of HBc VLPs with CFA/IFA resulted in the IgG2a/IgG1 ratios of 3.0 and 3.7 for intraperitoneal and subcutaneous injections, respectively. However, in mice immunised with Alhydrogel-adjuvanted HBc, this ratio was approximately 3.0 for both injections.

The lowest IgG2a/IgG1 ratio was found for HBc VLPs formulated in IFA or diluted in PBS; however, dependence on the immunisation route was also observed in both of these cases (Fig. 4B).

### Humoral and cell-mediated responses in mice after HBc-adjuvanting by Silica and Alhydrogel in combination with monophosphoryl lipid A

**Figure 5:**
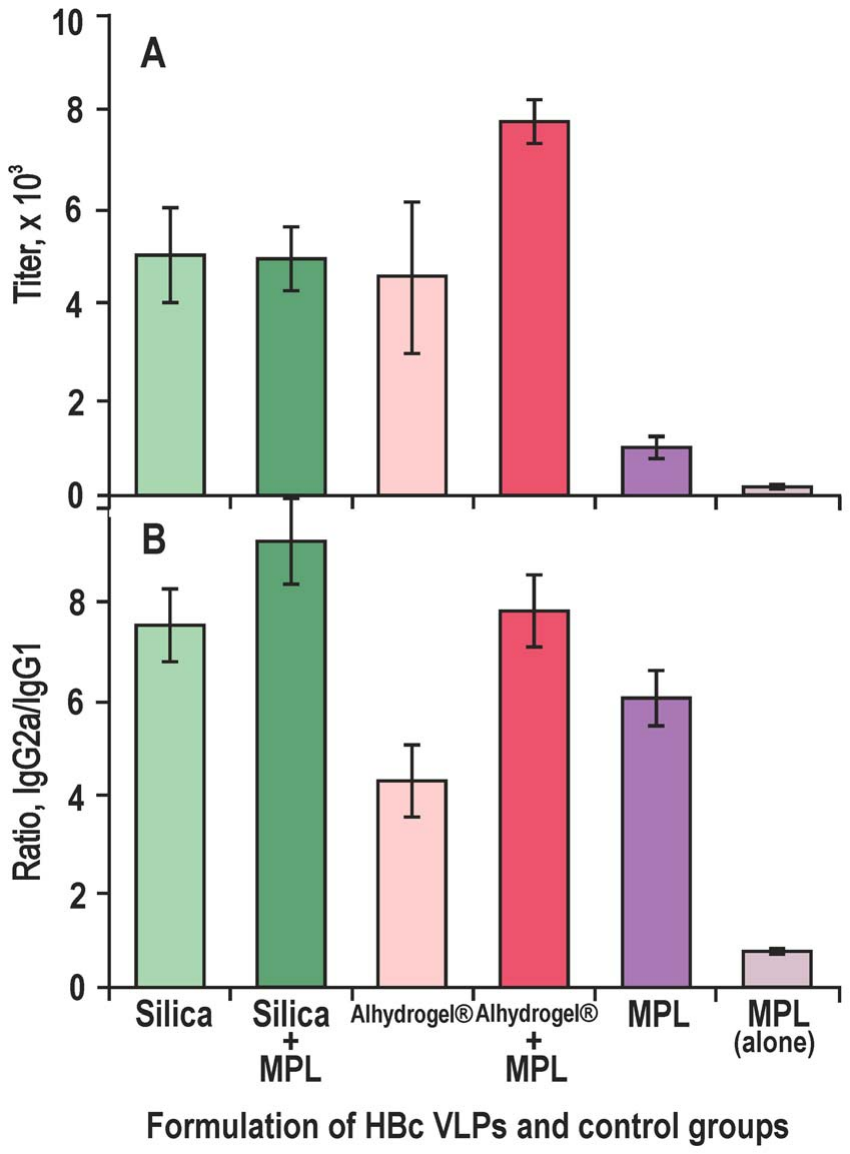
Induction of the humoral anti-HBc response in BALB/c mice by HBc VLPs formulated with different adjuvants in combination with MPL. **A** – total anti-HBc titers, **B** – ratio of anti-HBc IgG2a/IgG1 isotype titers. HBc VLPs were adjuvanted with Silica and Alhydrogel in the presence or absence of MPL or adjuvanted with MPL alone. Mice were bled after subcutaneous (s.c.) injection on day 10. The results represent anti-HBc titers or the ratios of the IgG2a/IgG1 titers as the means from five mice ± standard deviation (SD).

**Figure 6:**
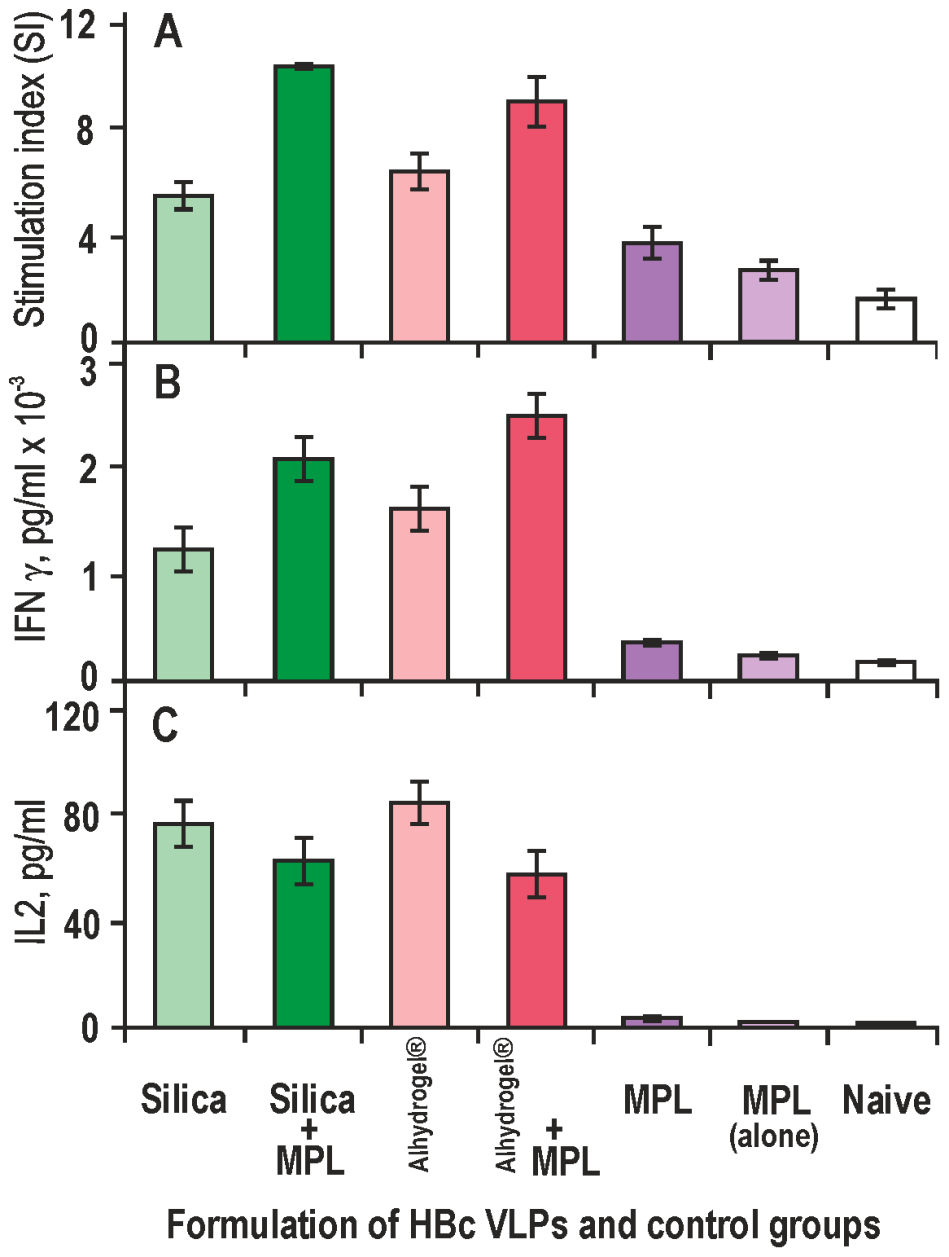
Proliferative response and cytokine production after immunisation of BALB/c mice with HBc VLPs formulated with different adjuvants in the presence or absence of MPL and splenocyte stimulation *in vitro*. **A** – Stimulation indexes (SI), **B** – interferon (IFN)-γ level in culture media, **C** – IL-2 level in culture media. Splenocytes were isolated at day 10 after subcutaneous immunisation and stimulated with HBc VLPs. SI values are presented as the mean stimulation indexes for triplicate wells ± standard deviation (SD). The cytokine production values are gained from duplicate wells ± SD. MPL (alone) – control group, mice immunised with 10 µg of MPL in PBS. Naive – control group or unstimulated *in vivo* mice.

To increase the induction of the cell-mediated immunity, HBc VLPs formulated with Silica or Alhydrogel were supplemented with MPL and both humoral (Fig. 5) and cell-mediated (Fig. 6) responses were evaluated in BALB/c mice on day 10 after immunisation. The MPL did no substantially increase the anti-HBc titer for the Silica-adjuvanted HBc VLPs and the anti-HBc levels were 1∶4,900 and 1∶4,500 for non-supplemented and supplemented formulations, respectively. The MPL supplement to Alhydrogel-adjuvanted HBc raised the anti-HBc titer from 1∶4,900 to 1∶7,700. The addition of MPL to the HBc VLPs in PBS, without any adjuvant, resulted in a low anti-HBc response of 1∶1,000 (Fig. 5A).

The IgG2a/IgG1 isotype ratios (Fig. 5B) were higher with MPL in both Silica- and Alhydrogel-adjuvanted HBc VLPs immunisations. Noticeably, the Silica-adjuvanted HBc VLPs immunisations with or without MPL provided higher IgG2a/IgG1 ratios than those of the appropriate Alhydrogel-adjuvanted immunisations.

The HBc-specific T-cell proliferation response (Fig. 6A) of mice splenocytes on day 10 after immunisation demonstrated MPL influence on cell activation; after the challenge of splenocytes with HBc VLPs *in vitro*, both Silica- and Alhydrogel-adjuvanted injections provided a definite increase in the T-cell proliferation as a result of the MPL contribution: 2.0 and 1.4 times, namely, SI = 11.0 and 9.5, respectively.

A slight increase of the stimulation index was observed in immunisation of mice with HBc VLPs in PBS (SI = 3.6) compared to immunisation with MPL alone (SI = 2.7) and splenocytes obtained from naive mice (SI = 1.9).

Because the Th1 and Th2 subsets of T-helper cells can be characterised by a specific pattern of cytokines produced, the levels of the Th1-characteristic IFN-γ (Fig. 6B) and IL-2 (Fig. 6C) cytokines were measured in response to the stimulation of splenocytes with HBc VLPs *in vitro*. While the level of IFN-γ was undoubtedly enhanced, the level of IL-2 synthesis did not demonstrate any increase as the result of the MPL addition to Silica- and Alhydrogel-adjuvanted HBc VLPs.

## Discussion

The main goal of the present study was the evaluation of the adjuvant properties of the commercially available Silica nanopowder, with an original particle size of 10–20 nm, to provide a putative vaccine with high humoral and cell-mediated immune response in comparison to the traditional Alum, CFA and IFA adjuvants.

The role of the biological component of the vaccine is assigned here to HBc VLPs, which belong to the most studied VLP carriers and appear as a basis for a long list of promising vaccines against hepatitis A and B, anthrax, influenza, malaria, borreliosis, cattle theileriosis, infectious bursal and many other diseases (for a review see [3], [23], [25], [26]). The HBc particles are also distinguished by their extremely high intrinsic immunogenicity in hepatitis B patients [37], their capability to function as both T-cell-dependent and T-cell-independent antigens [38], and by their ability to induce not only strong B-cell but also remarkably strong T-helper cell and CTL responses [39]–[41]. HBc VLPs formed by the full-length HBc protein are able to prefer the Th1 response over the Th2 response and induce high levels of IgG2a antibodies [24], [36]. Lastly, the HBc VLPs are unique among known VLP models due to their ability to undergo recognition and processing by B cells rather than by professional non-B antigen-presenting cells, such as macrophages or dendritic cells [42], [43].

The critical question of this study was directed at the ability of non-modified Silica particles to bind HBc VLPs because most of the previous reports were based on the application of mesoporous Silica [16], [18]–[21]. The cationisation of Silica with the formation of Silica-based cationic bilayers was also reported as a prospective way to improve adsorption of the biological component [44]. In contrast to a recent study [22], which did not reveal any adsorption of the murine polyomavirus-like particle capsomeres to non-modified Silica particles, we demonstrate a notable adsorption level of HBc VLPs to the commercially prepared Silica nanoparticles that was evidenced by DLS (Fig. 1), spectrophotometry (Fig. 2), and electron microscopy (Fig. 3).

As for the binding efficiency of the biological component, the Silica exhibits a definite equilibrium at the level of 30–40% of the adsorbed HBc VLPs, with a low correlation with the Silica concentration in the formulation mixture (Fig. 2). This is in contrast to the Alhydrogel-adjuvanted HBc VLPs, were a strong correlation of the adsorption with the adjuvant concentration was observed with almost total binding (92%) at the highest Alhydrogel concentration used here. This observation is important for the possible toxicity and safety reasons (see below) because it allows to use a decreased dose of the Silica adjuvant for the immunisation.

To formulate a vaccine, the 2 h incubation time has been chosen on the basis of our preliminary experiments, to establish full equilibrium in the formulation mixture. It is necessary to keep in mind that protein adsorption is a very fast process, e.g., the adsorption of a large group of different representative proteins to the Alum adjuvant has been shown to occur within one minute; however, at least 1 h of incubation and regular mixing is necessary for the uniform surface coverage of the adjuvant by protein [45].

The obvious difference in the HBc VLP binding to Silica and Alhydrogel had, however, no negative impact on the level of immune response to HBc VLPs, as we have shown here. While the strength of the initial adsorption of the biological component to the Alum adjuvant in a vaccine is highly important, according to the data in the literature, the strength of the immune response correlates more with the degree of adsorption of the antigen to the particular adjuvant in the interstitial fluid of immunised animals [46]–[48].

After the binding efficiency, the next critical question of the formulation process relates to the size of the aggregates formed by the adjuvant and the biological component. It is known that original 2-nm particles of Alum may form stable porous aggregates with diameters of 1-10 µm [49]. According to our observations, both Silica and Alhydrogel-adjuvanted materials form structures larger than 10 µm in size, which sediment efficiently during low-speed centrifugation. We hypothesise that Silica nanoparticles suspended in PBS form quasi-porous structures, which are similar to those of mesoporous Silica, and this formation therefore enables the good adsorption of the HBc VLPs within the emerging “holes” between the initial small nanoparticles in the final aggregate. This suggestion is indirectly confirmed by a recent finding that non-porous Silica particles of 50 nm in size, but not those of 1 µm in diameter, were efficient in supporting the immunological response to the capsomeres of murine polyomavirus-like particles [22].

Concerning the role of injection routes, the Silica-adjuvanted, like the CFA/IFA- and IFA-adjuvanted HBc VLPs, demonstrate equally high humoral responses by both intraperitoneal and subcutaneous immunisation, which is in opposition to the Alhydrogel-adjuvanted antigen, where subcutaneous immunisation appears less efficient (Fig. 4A). The latter is consistent with a previous observation that Alum-adjuvanted immunisation leads to a higher antibody response by intraperitoneal rather than by the subcutaneous administration [50]. One possible explanation of this finding could be connected to the Alum-induced local inflammatory response, irritation, and itching reactions, when the inoculum is not quickly dispersed from the subcutaneous injection site [51].

The next critical point in the adjuvant application accounts for its role in the spread among the Th1 and Th2 response types. Fig. 5B shows that Silica as an adjuvant may advance the intrinsic property of the HBc VLPs to be processed by Th1 cells with preferred production of IgG2a isotype antibodies. The latter are preferable for potential vaccines because a Th2-biased immune response with production of IgG1 antibodies is generally not as effective as needed to protect mice, for example, against hantavirus challenge [52]. Moreover, our experiments show that the combination of Silica nanoparticles with MPL leads to a definite improvement in the T-cell response to the HBc VLPs, as follows from the appropriate stimulation indexes of splenocytes (Fig. 6A) and the levels of induced cytokines, particularly the level of the interferon-γ (Fig. 6B).

The problem of the potential toxicity of the Silica adjuvant is of the utmost importance. The biological toxicity of the Silica nanoparticles may be connected, first, to the intratracheal instillation, which could develop and exacerbate both the airway hyper-responsiveness and airway remoulding [53]. In this case, the particles, less than 200 nm in diameter, could be more cytotoxic than the larger particles of the same material [54], [55]. Thus, the formation of large aggregates by the Silica-adjuvanted HBc VLPs, which is demonstrated in the present study, provides a definite advantage for the proposed methodology.

Therefore, our findings present a new prospective adjuvant for the delivery of virus-like particles. We show for the first time that HBc VLPs can be adsorbed on commercially available Silica nanoparticles with the initial size of 10–20 nm forming large aggregates, larger than 1 µm, after formulation. These Silica nanoparticles-HBc VLPs complexes are able to induce strong Th1-biased immune responses in mice.

Noteworthy, SiO_2_@LDH, which is comprised of a core-shell nanoparticle with a mesoporous Silica as the core and layered double hydroxides (LDH) as a shell, with a diameter of approximately 210 nm, was proposed recently as a promising adjuvant for the delivery of a DNA vaccine [56].
